# Circular RNA in Cardiovascular Diseases: Biogenesis, Function and Application

**DOI:** 10.3390/biom14080952

**Published:** 2024-08-06

**Authors:** Shuai Mei, Xiaozhu Ma, Li Zhou, Qidamugai Wuyun, Ziyang Cai, Jiangtao Yan, Hu Ding

**Affiliations:** 1 Division of Cardiology, Departments of Internal Medicine, Tongji Hospital, Tongji Medical College, Huazhong University of Science and Technology, 1095# Jiefang Ave., Wuhan 430030, China; moshine@hust.edu.cn (S.M.); xzma2023@hust.edu.cn (X.M.); m202276233@hust.edu.cn (L.Z.); d202282253@hust.edu.cn (Q.W.); m202376476@hust.edu.cn (Z.C.); 2 Hubei Key Laboratory of Genetics and Molecular Mechanisms of Cardiological Disorders, Wuhan 430030, China; 3 Key Laboratory of Vascular Aging, Ministry of Education, Tongji Hospital, Tongji Medical College, Huazhong University of Science and Technology, 1095# Jiefang Ave., Wuhan 430030, China

**Keywords:** cardiovascular disease, circular RNA, biogenesis, degradation, clinical applications

## Abstract

Cardiovascular diseases pose a significant public health challenge globally, necessitating the development of effective treatments to mitigate the risk of cardiovascular diseases. Recently, circular RNAs (circRNAs), a novel class of non-coding RNAs, have been recognized for their role in cardiovascular disease. Aberrant expression of circRNAs is closely linked with changes in various cellular and pathophysiological processes within the cardiovascular system, including metabolism, proliferation, stress response, and cell death. Functionally, circRNAs serve multiple roles, such as acting as a microRNA sponge, providing scaffolds for proteins, and participating in protein translation. Owing to their unique properties, circRNAs may represent a promising biomarker for predicting disease progression and a potential target for cardiovascular drug development. This review comprehensively examines the properties, biogenesis, and potential mechanisms of circRNAs, enhancing understanding of their role in the pathophysiological processes impacting cardiovascular disease. Furthermore, the prospective clinical applications of circRNAs in the diagnosis, prognosis, and treatment of cardiovascular disease are addressed.

## 1. Introduction

Cardiovascular diseases (CVDs) are the leading cause of mortality worldwide, imposing a significant medical burden, particularly in developing countries, where they account for 40% of deaths [1,2,3]. The incidence of CVDs is affected by multiple factors, with age and chronic conditions being paramount as life expectancy increases [1,4]. By 2030, approximately 20% of the population will be 65 years or older, making CVDs responsible for 40% of all deaths and therefore the predominant cause of mortality [5]. Chronic diseases, such as diabetes mellitus and hypertension, are critical factors in both the incidence and prevalence of CVDs, exacerbating the situation due to demographic growth, aging, and unhealthy lifestyles [6,7]. Furthermore, underdiagnosis, undertreatment, or inadequate treatment of chronic conditions amplify the severity of the issue [8]. Therefore, it is imperative to develop effective therapeutic targets to reduce cardiovascular risk and manage CVDs.

Circular RNAs (circRNAs) represent a novel class of non-coding RNAs, characterized by their covalently closed loop structure, which results from back-splicing of the 5′ cap and 3′-terminal of precursor RNA. Although initially discovered in the 1970s [9], circRNAs initially received minimal attention from researchers and were believed to arise from “missplicing” or “scrambled splicing” events [10]. In the 2010s, advanced sequencing technologies facilitated the identification of large numbers of circRNAs in mammalian tissues, including those of humans, mice, rats, pigs, and monkeys, with more than 140,790 circRNAs cataloged in human tissues and cells [11]. Subsequent studies have demonstrated that circRNAs play crucial roles in growth, development, and disease processes by regulating gene expression through various mechanisms, including acting as microRNA (miRNA) sponges, interacting with proteins, influencing gene transcription, and affecting translation. Despite growing interest, the specific roles of circRNAs in CVDs remain poorly understood, and their regulatory mechanisms are still largely unexplored. This review aims to provide a comprehensive overview of the properties, biogenesis, functions, and applications of circRNAs in CVDs.

## 2. Characteristics of CircRNAs

CircRNAs have a unique covalently closed structure that lack free ends, such as the 5′ cap and 3′ tail, making them more stable and resistant to digestion by exoribonucleases like Rnase R and RNase D than linear RNAs [12,13]. Furthermore, the absence of a poly(A) tail in circRNAs prevents reverse transcription by oligo primers, necessitating the use of random primers, whereas messenger RNA can undergo reverse transcription using both types of primers [14]. Furthermore, the junction site sequence of circRNAs can be determined through Sanger sequencing. In particular, due to gene rearrangement, upstream sequences may integrate into downstream sequences, creating sites that mimic circRNA junction sites. To mitigate this problem, researchers typically employ divergent and convergent primers to amplify DNA fragments at genomic DNA and cDNA levels through polymerase chain reaction (PCR). In genomic DNA, divergent primers are unable to amplify DNA fragments, which can be achieved with convergent primers, and both types of primers can amplify DNA fragments in cDNA. These methods are widely used in studies to confirm the presence of circRNAs in cells.

CircRNAs are widely distributed in various tissues of mammals. Sequencing data from multiple tissues and cells indicate that circRNAs are abundant and prevalent in almost every cell type and tissue. Interestingly, the abundance of circRNAs does not necessarily correlate with the expression of their parental genes in cells or tissues, suggesting potential functional significance. For example, circHIPK3, one of the most abundant circRNAs, is widely present in the heart [15,16], intestine [17], stomach [18], lung [19], and other tissues. CircRNAs also exhibit spatial and temporal specificity; a particular circRNA may exist exclusively in a specific cell type or tissue and may vary across different stages of development and disease conditions. Furthermore, circRNAs derived from the same parental gene can have multiple subtypes [20]. For example, at least seven distinct circRNAs are produced from the human *CAMSAP1* gene [21]. The alternative splicing leading to the formation of these subtypes can be observed at many gene loci, indicating that alternative circularization is a widespread phenomenon in circRNA biology. These observations suggest that the expression of circRNA during growth, development, and disease progression is flexible and complex [22].

Based on sequence composition, circRNAs are classified into three types: exon type (Exonic circRNA, EcircRNA), intron type (Circular intronic RNA, ciRNA), and mixed type exon–intron (Exon–intron circRNA, EIciRNA) [23]. A great number of highly expressed EciRNAs comprise the middle exons of coding genes and typically contain two to three exons [21]. Generally, EciRNA with only one exon is considerably longer than that formed from multiple exons [21]. As EciRNAs are constituted by exons, they exhibit higher species conservation, akin to protein-coding genes, than long non-coding RNAs. EciRNAs are predominantly located in the cytoplasm [12]. In contrast, ciRNAs tend to be more abundant and broadly expressed in human cells. For example, in HeLa cells, 37,277 new ciRNAs were identified, compared with only 1374 EcircRNAs. Similarly, in C2C12 cells, 16,768 new ciRNAs were identified compared with 573 EcircRNAs [24]. CiRNAs are primarily distributed in the nucleus, a characteristic that contrasts with their counterpart mRNAs that are mainly found in the cytoplasm [25]. Except for this property, ciRNAs are much less evolutionarily conserved compared with EcircRNAs, due to lack of the same consensus RNA elements at their branchpoint sites [25].

## 3. Biogenesis of CircRNAs

CircRNAs are generated during the RNA splicing process, which can be regulated by functional elements and multiple factors. Previous studies on circRNA formation have identified four regulatory models: flanking inverted-repeat sequences, RNA-binding proteins (RBPs), lasso structures, and m6A modification.

### 3.1. Flanking Inverted-Repeat Sequences

**Figure 1:**
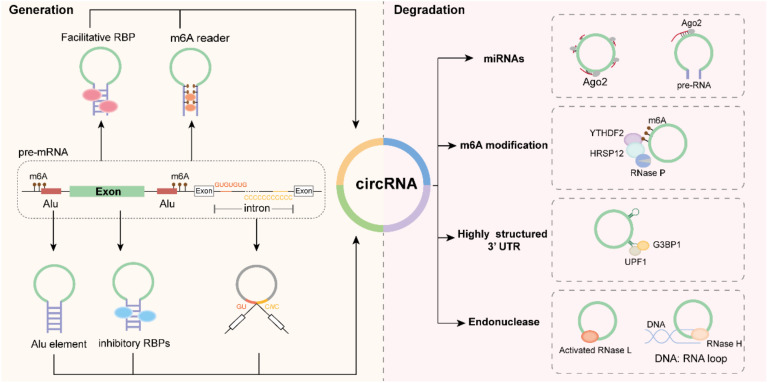
The biogenesis and degradation of circRNAs. Left. The biogenesis of circular RNA was regulated by Alu element, RNA binding proteins, m6A modification and lariats. Right, the degradation of circular RNA was modulated by miRNAs, m6A modification highly structured 3′UTR and Endonuclease.

In the human genome, short interspersed elements (SINEs) and long interspersed elements (LINEs), prevalent in intron sequences, represent the most common classes of retrotransposable elements. Among these, Alu elements, which constitute over 10% of the human genome, are the dominant type of human SINEs [26,27]. The production of circRNAs induced by Alu elements in flanking inverted-repeat sequences is notably common in EciRNAs. In humans, 88% of circRNAs contain Alu elements in their flanking introns, which are likely to facilitate circularization [28]. During pre-RNA splicing, Alu elements in the flanking introns can interact complementarily, bringing the two ends spatially closer and ultimately promoting the formation of circRNAs composed of exons [29,30,31] (Figure 1). CircRNA ciRS-7 (CDR1as), an extensively studied circRNA, has been confirmed to be generated through Alu elements via back-splicing and CRISPR/Cas9-mediated deletion assays [31]. Moreover, several other Alu element-dependent circRNAs have been identified through computational analysis and experimental approaches, supporting the conclusion that Alu element-mediated RNA circularization is a prevalent biosynthesis method for circRNAs [29,30,31]. Additionally, the conservation of circRNA biogenesis between mice and humans was examined using 71 circRNAs present in both species, revealing that these conserved circRNAs share similar biogenesis mechanisms [28,32].

### 3.2. RBPs

RBPs serve as a crucial transregulatory element in the formation of EciRNAs. More than a dozen RBPs have been identified as regulators of circRNA synthesis. These proteins typically bind to flanking inverted-repeat sequences, either promoting or unwinding double-stranded nucleotides (Figure 1). Based on their effect on circRNA biogenesis, RBPs are categorized as inhibitory or facilitative. DExH-Box helicase 9 (DHX9), a prevalent nuclear RNA helicase, is classified as an inhibitory RBP [33]. CLIP sequencing analysis of DHX9 shows that it predominantly interacts with intronic RNA and that the DHX9 peaks are located primarily in Alu SINEs. Approximately 60% of the DHX9 peaks occur on or within 100 nucleotides of Alu repeats, in contrast to only 3–10% in controls. Reduction of the DHX9 protein enhances circRNA production and impairs translation. DHX9 is involved in the biogenesis of various circRNAs, including circRNA-CREIT [34], circPDIA4 [35], circBNC2 [36], cSMARCA5 [30], and circPICALM [37]. Another RBP, adenosine deaminases acting on RNA 1 (ADAR1), a double-stranded RNA editing enzyme, acts as a suppressor of circRNA production [28]. ADAR1 affects circRNA biogenesis by mediating the A to I modification at ALU elements [38]. ADAR1 knockdown significantly and specifically increases circRNA expression [22,39,40].

Facilitative RBPs, such as Quaking (QKI), FUS [41], and mannose-binding lectin (MBL), promote the generation of circRNAs during the RNA splicing process. Conn et al. demonstrated that more than one-third of the abundant circRNAs is dynamically regulated by QKI during the human epithelial–mesenchymal transition (EMT). And intronic QKI binding motifs are sufficient to induce circRNA formation during the splice event [42]. In the cases of doxorubicin-mediated heart failure, Qki promotes the biogenesis of multiple circRNAs, such as circTtn, circFhod3, and circStrn3, mitigating the effects of doxorubicin-induced cardiac apoptosis and atrophy and ultimately enhancing cardiac function [43]. Furthermore, in metabolic diseases, Qki facilitates the formation of circRNA circGlis3, improving β-cell apoptosis and islet β-cell dysfunction in obesity [44]. FUS, a prion-like protein containing intrinsically disordered domains, combines with several splicing factors to form FUS interactors that participate in abnormal RNA metabolism in the neurodegenerative disease amyotrophic lateral sclerosis (ALS) [45]. Similarly to other facilitative RBPs, the FUS function that regulates circRNA biogenesis also binds to the ALU element that flanks the backsplicing junctions [41]. CircRNA (circMbl) is derived from the second exon of the splicing factor *muscleblind* (*MBL*), and its flanking introns contain a conserved binding motif, which is strongly and specifically bound by its parent gene *MBL*. Dynamic changes in MBL levels strongly affect circMbl biosynthesis, which is dependent on MBL binding sites [46]. Other RNA binding proteins, including KRAS [47], ZEB1 [48], ESRP1 [49], RIG-I [50], and EIF4A3 [51,52], also participate in circRNA biogenesis.

### 3.3. Lariat Precursor Construction

Lariat structures often play a key regulatory role in the formation of intronic circRNAs. Lariats are formed by intronic sequence during RNA splicing, involving a 2′-5′ phosphodiester linkage at a branch point. The production of ciRNA involves a consensus motif that contains 7nt GU-rich elements near the 5′ segment and 11nt C-rich elements near the branch point in ciRNA-producing introns. During the generation of ciRNA, these two elements combine to form a lariat structure and are catalyzed by debranching enzymes (Figure 1). Ci-ankrd52 represents the first identified ciRNA emerging from the interaction of these components [25,53]. This lariat architecture is rare among other types of circRNA, underscoring its pivotal role in the generation of ciRNA.

### 3.4. m6A-Mediated CircRNA Biosynthesis

RNA methylation involves the addition of methyl groups to specific nucleotide residues in RNA, such as N1-methyladenosine (m1A), 7-methylguanosine (m7G), 5-methylcytosine (m5C), N6-methyladenosine (m6A), or 6,20-O-dimethyladenosine (m6Am) [54,55]. Among these, the m6A modification has been the most extensively studied, and significantly impacts on RNA processing, splicing, translation, and stability [56]. Zhou et al. developed a computational pipeline (AutoCirc) to define thousands of m6A circRNAs using m6A RIP-seq and defined the m6A modifications map on circRNAs. This analysis demonstrates that m6A circRNAs are frequently derived from exons that are not methylated in mRNAs [57]. The biogenesis of circ-ZNF609 is influenced by m6A modification, as shown by two engineered vectors, p-circ and mutant-circ. P-circ facilitates the production of circ-ZNF609 in the absence of its natural flanking introns, whereas mutant-circ, which features mutations at the m6A site, exhibits a significant decrease in circRNA levels with a concurrent increase in precursor RNA [58]. In rhabdomyosarcoma, DDX5 and YTHDC1 directly promote the upregulation of a subset of circRNAs by recognizing m6A modification [59]. The biogenesis of circRNA circDDIT4 is also largely facilitated by m6A modification, with the WTAP/METTL3/METTL14 methyltransferase complex promoting its expression, whereas the RNA demethylase FTO decreases expression [60]. Therefore, the formation of circRNAs is associated with m6A recognition proteins, which could be recruited by the m6A site in the intron region and then promote the spatial proximity of the splicing sites [61] (Figure 1).

## 4. Degradation of CircRNAs

As mentioned above, circRNAs exhibit enhanced stability and resilience compared with linear RNAs because of their unique structure. Consequently, they are subjected to distinct and specialized degradation mechanisms. Drawing from pertinent studies, we have classified these processes into five categories: miRNA-mediated degradation, m6A modification-mediated degradation, exonuclease-mediated degradation, UPF1/G3BP1-mediated structure-dependent degradation, and GW182-mediated degradation.

### 4.1. miRNA-Mediated Degradation

Recent studies have indicated that miRNAs could be involved in circRNA degradation mediated by Argonaute2 (Ago2), an active slicer enzyme (Figure 1). CircRNA CDR1as has a remarkable capacity to bind to multiple miRNAs. In particular, miR-671 can form a double-stranded structure with CDR1as. Subsequently, Ago2 recognizes and cleaves this double-stranded structure, leading to the degradation of circRNA [62]. In the spinal nucleus, circRNA-Filip1l sponges miRNA-1224, forming an RNA-RNA complex that is degraded in an Ago2-dependent manner, ultimately leading to a reduction in mature circRNA-Filip1l [63]. These findings illustrate that miRNA-mediated circRNA degradation comprises a novel, biologically relevant, and evolutionarily conserved regulatory mechanism.

### 4.2. m6A Modification-Mediated Degradation

N6-methyladenosine (m6A) methylation is the most prevalent epigenetic modification, regulating RNA stability and degradation. The first report of circRNA degradation mediated by m6A modification was by Park et al. in 2019. Their work showed that YTHDF2 could recognize and bind to the circRNA m6A modification site, then recruit the HRSP12 protein (adaptor protein), which serves as a bridge to connect the endoribonucleases RNase P/MRP complex and YTHDF2, and eventually results in the RNA degradation process [64] (Figure 1). The subsequent cancer study displays a circRNA circ3823 degraded by YTHDF3 and ALKBH5 cooperates with YTHDF2 in colorectal cancer and may serve as a novel target for treatment of CRC [65]. In gastric cancer, silencing of METTL14 expression significantly reduced the m6A levels of circORC5, but increased circORC5 expression, associated with a poor survival [66].

### 4.3. Exonuclease-Mediated Degradation

Due to their special covalent structure, circRNAs are resistant to degradation by exonucleases, which typically digest nucleic acids from the 5′ to 3′ end. Nevertheless, circRNAs can be cleaved at specific sequences by endonucleases and can mediate the degradation of circRNAs, particularly in immune-related conditions, such as viral infections and innate immunity. Chen et al. discovered that circRNAs are globally degraded by RNase L when cells are stimulated with poly(I:C) or during viral infections. This degradation process can be activated by free PKR in the early stages of the innate cellular immune response [67,68]. Additionally, another endonuclease, RNase H1, is involved in circRNA degradation by recognizing and cutting DNA:RNA hybrids (R-loops) (Figure 1). Ci-ankrd52 is prone to form an R-loop with DNA compared with its cognate pre-mRNA. Therefore, during the transcriptional process, ci-ankrd52 is likely to bind to DNA and competitively promote the release of premRNA from R loops, followed by degradation by RNase H1, to promote efficient transcriptional elongation [69]. 

### 4.4. UPF1/G3BP1-Mediated Structure-Dependent Degradation 

The 3′ untranslated region (3′ UTR) harbors specific attributes that guide RNA decay, predominantly orchestrated by UPF1 and G3BP1, two pivotal RBPs. It is estimated that approximately one-third of circRNAs in humans are capable of adopting highly structured configurations. According to a theoretical model, UPF1 attaches to these structured RNAs, whereas G3BP1 interacts with UPF1, contributing to the RNA degradation pathway. Consequently, researchers have initiated studies to determine whether the UPF1/G3BP1 complex influences the degradation of circRNAs. Depletion of either protein results in elevated levels of mRNAs and circRNAs containing complex structures [70]. These observations imply that intricate structures of circRNAs can be subjected to degradation through the UPF1/G3BP1 pathway (Figure 1).

### 4.5. GW182-Mediated Degradation

The protein *Drosophila* GW182 and its human counterparts TNRC6A, TNRC6B, and TNRC6C are key components of the P-body and the RNAi machine. They help in assembling the P-body and act as a molecular scaffold connecting RNA-induced silencing complexes and various mRNA decay enzymes [71,72]. When GW182 or its human counterparts are silenced, there is a significant increase in circRNAs in both drosophila and humans, indicating the important role of GW182 in circRNA degradation. Further investigation revealed that the middle domain of GW182 is dispensable for circRNA degradation. However, silencing of other P-body components or RNAi machine factors has no effect on circRNA levels, indicating the critical role of GW182 and its human counterparts in circRNA degradation [73]. 

## 5. Biological Functions of CircRNAs

As a new type of non-coding RNA, circRNAs play a key role in the whole process of gene expression, including DNA modification, RNA transcription, post-transcriptional and protein regulation. Through in-depth research, the regulatory effects of circRNA can be divided into the following categories: miRNA sponge, binding to protein complex, translating proteins, modulating translation process, regulating gene transcription, and competing with parental genes. 

### 5.1. miRNA Sponge

**Figure 2:**
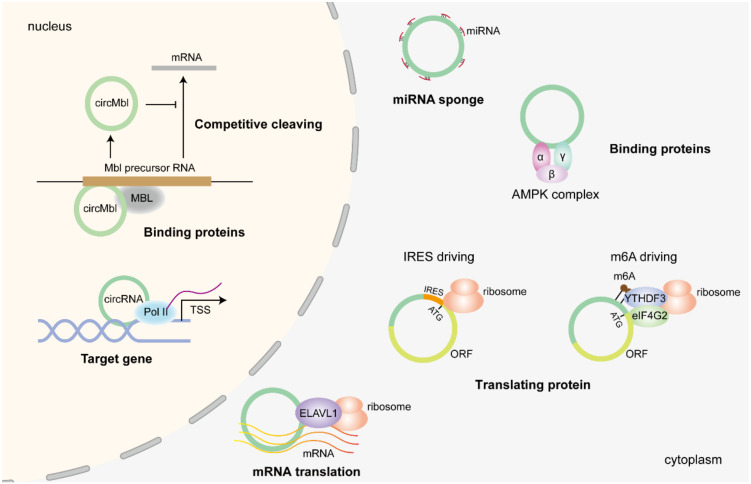
The function of circRNA. In cytoplasm, circular RNA could act as a miRNA sponge, bind to proteins, and translate proteins; in the nucleus, circular RNA could modulate the transcriptional process and participate in RNA splicing.

miRNA sponges represent the earliest and most extensively studied function of circRNAs, predominantly occurring in the cytoplasm (Figure 2). The first circRNA to be investigated, ciRS-7, contains more than 70 conserved miRNA binding sites [74]. Of these, miR-7 displays the strongest affinity for binding. ciRS-7 counteracts the suppressive effect of miR-7 on its target genes and plays a role in the regulation of the central nervous system by binding through base complementary pairing. In addition, circRNA circSry also possesses multiple miRNA binding sites acting as a miRNA sponge. To date, an increasing number of circRNAs have been found to have many miRNA binding sites and can serve as competitive endogenous RNAs (ceRNAs), such as circHIPK3 [29,75,76], and circPVT1 [77]. Although many studies have elucidated the role of circRNA as a miRNA sponge, some researchers have questioned the effectiveness of circRNAs as miRNA sponges, arguing that changes in circRNAs are generally insufficient to reverse miRNA-mediated suppression due to sparse binding sites or low abundance [78]. Therefore, specific stoichiometric conditions must be considered and achieved for circRNAs to function effectively as miRNA sponges [79,80,81]. Therefore, the consensus is that circRNAs with high-abundance and a large number of miRNA binding sites are more likely to be effective as a sponge.

Despite numerous studies aiming to illustrate the role of circRNA in sponges in diseases, some studies have shown a positive correlation between the expression of circRNA and miRNA levels, such as circCSNK1G3 and miR-181b/d. Knockdown of circCSNK1G3 expression decreased the levels of miR181b/d, while the overexpression of circCSNK1G3 increased. Furthermore, a similar relationship also exists in circRNA Cdr1as and miR-7, which is not a traditional “sponge” relationship [62]. These findings suggest that there is a dynamic equilibrium in circRNAs and miRNAs. However, the specific mechanism has not been fully explored.

### 5.2. Binding Proteins

As a direct effector of biological functions, proteins significantly alter the function of organisms and influence pathophysiological processes. Therefore, the underlying mechanism of circRNA-binding proteins has attracted great attention from researchers in various disease processes (Figure 2). In this section, according to the effects of circRNA on proteins, the modulating patterns of circRNAs are classified into four categories: protein decoy; protein scaffold; protein stabilizers; and protein degradation promotor. CircMbl is circularized by the second exon of the MBL gene and recruits the MBL protein to participate in the MBL precursor RNA splicing process, and competitively inhibits the production of messenger RNA [46]. Recent studies have shown that many circRNAs can bind proteins: circACC1, a circRNA derived from the lipid metabolism-related gene *ACC1*, can bind to the β and γ subunits of AMPK, maintain the stability of the AMPK complex, promote downstream ACC1 and PKM phosphorylation by AMPK, and participate in the energy metabolism process in cancer [82]. CircRNA circ-Ccnb1, derived from the cell proliferation-related gene *Ccnb1*, can form a triplet complex with Ccnb1 and CDK1, block the function of Ccnb1 and CDK1, and inhibit the activities of tumor cells [83]. CircFOXP1 can interact with PTBP1, inhibit the 3′ UTR and CDS regions of PTBP1-binding PKLR mRNA, promote the expression of PKLR, and accelerate the appearance and development of gallbladder cancer [84].

### 5.3. Translating Proteins

#### 5.3.1. Internal Ribosome Entry Site (IRES) Driving circRNA Translation

As a cis-acting RNA element, IRES promotes protein translation in a 5′-cap-independent manner [85]. Recently, studies have demonstrated that circRNAs have the potential to translate protein independent of the 5′ cap structure, one of which involves IRESs (Figure 2). Chen et al. applied the oligo-split-eGFP circRNA reporter to systematically identify IRES activity that facilitates circRNA translation, which is mediated by Watson–Crick base-pairing between IRES and 18S ribosomal RNA (18S rRNA). They also identified a novel circRNA-encoded protein, circFGFR1p, which suppresses cell proliferation by negatively regulating FGFR1 under stress conditions [86]. Circ-ZNF609 is highly expressed in myofibers, which can encode polypeptides and promote the proliferation of myofibers [87]. In addition, Zhang et al., focusing on encoding proteins of circRNAs, found that the peptide FBXW7-185aa encoded by circ-FBXW7 can antagonize USP28-regulated c-myc stability, promote the degradation of c-myc, and inhibit glioma progression [88]. The AKT3-174aa peptide, encoded by circ-AKT3 expressed in glioblastoma, can bind to PDK1 and reduce threonine phosphorylation at AKT308, thus participating in the inhibition process of glioblastoma [89]. Furthermore, circ-HER2 [90], circYAP [91] can also be translated through IRES. These studies demonstrate a novel regulatory mechanism of circRNAs.

#### 5.3.2. N6-Methyladenosine (m6A) Driving circRNA Translation

The m6A-dependent translation process is another critical mechanism of circRNA translation. M6A RIP-seq data have shown that m6A-modified circRNAs with coding potential are prevalent in human tissues and cells [92,93]. Wang et al. found that the m6A reader YTHDF3 bound to the m6A modification site of circRNA and recruited eIF4G2 and other translation initiation factors to promote the translation of circRNA [94,95] (Figure 2). CircRNA circ-ZNF609, circ-YAP follows this pattern to translate its peptide [58]. In hepatocellular carcinoma, the m6A reader IGF2BP1 recognized the site on the circRNA circMAP3K4 and initiated the translation process. Another m6A reader, YTHDF2, facilitated the production of 404-amino-acid MET variant (MET404) encoded by circular MET (circMET) [96]. A newly identified oncogenic circRNA circARHGAP35 has an m6A-modified site at the start codon of the open reading frame and encodes a truncated protein that promotes oncogene activation in cancer [97].

### 5.4. Mediating mRNA Translation

In the translation process, circRNA could also participate in mRNA translation, which occurs mainly in the nucleus and often occurs in circRNA containing introns (Figure 2). The translational efficiency of FLT3 kinase could be improved by increasing the binding of polypyrimidine tract-binding protein 1 (PTBP1) to FLT3 messenger RNA, which is mediated by circMYBL2 [98]. CircRNA circZNF609 recruited the RBP ELAVL1 and then directly interacted with the mRNA of CKAP5, UPF2, and SRRM1, increasing their stability and translation, which, in turn, regulated microtubule function in cancer cells and sustained cell cycle progression [99].

CircRNAs not only promote the translation process, but also exert a critical inhibitory effect on mRNA translation. CircMALAT1 prevents the binding between ribosomes and PAXS mRNA by binding to PAXS mRNA, thus suppressing the level of PAXS protein and promoting hepatoma cells [100]. CircPDE5A disrupts the translation of eukaryotic translation initiation factor 3c (EIF3C) mRNA by blocking m6A methylation of EIF3C mRNA [101]. CircVAMP3 modulates phase separation of CAPRIN1, which inhibits c-Myc mRNA translation, and eventually negatively regulates the proliferation and metastasis of HCC cells in cancer [102].

### 5.5. Regulation of Gene Transcription

CircRNAs are also involved in the regulation of gene transcription in the nucleus (Figure 2). CircRNAs circEIF3J and circPAIP2, composed of exons and introns, can recruit Pol II and U1 snRNP to form transcriptional complexes by binding to the promoter region of genes and participating in the regulation of downstream genes [103]. Ci-ankrd52 interacts with Pol II machinery and positively regulates Pol II transcription [25]. Furthermore, HIV-induced circRNA ciTRAN decoys serine/arginine-rich splicing factor 1 (SRSF1) from the viral transcriptional complex, thus promoting efficient viral transcription [104]. In addition, exon-type circRNAs in the nucleus participate in gene regulation by regulating transcription factors. Circ-HuR binds to cellular nucleic acid-binding protein (CNBP) and inhibits the interaction between CNBP and the promoter region of the HuR gene, thus reducing HuR expression and tumor progression [105]. CircSMARCA5 interacts with its parent gene locus to form an R loop, stopping the transcriptional process at exon 15 of *SMARCA5*, a mechanism which eventually improves sensitivity to cytotoxic drugs in breast cancer [106].

### 5.6. Competitive Cleaving with Parental Messenger RNA

Although relatively rare, understanding the regulatory mechanism of circRNAs is necessary to broaden our considerations of circRNA (Figure 2). To investigate the relationship between circRNA biogenesis and canonical splicing, researchers conducted a fly model carrying a variant of the large subunit of RNA polymerase II, which has been reported to increase cotranscriptional splicing efficiency. By analyzing total and circRNA-enriched RNA-seq data, they found that flies with the mutation had significantly lower circRNA levels. Next, they constructed minigenes that carry circRNA exons and flanking introns to generate circRNAs efficiently. However, they discovered that flanking exons possessing strong 5′ and 3′ splice sites significantly decreased circularization efficiency. Those results revealed a strong competition between exon circularization and canonical splicing [46]. They also identified a circRNA circMbl that enhances the binding intensity of MBL to the exon 2 region of the Mbl precursor RNA, promoting its own expression and inhibiting the expression of mature mRNA [46].

## 6. CircRNAs and CVDs

### 6.1. Atherosclerosis

Atherosclerosis is an important basis for the pathogenesis of cardiovascular and cerebrovascular diseases, which affects patient survival and prognosis [107]. Current research is directed toward deciphering the regulatory mechanisms of atherosclerosis through the lens of circRNA. Genome-wide association studies (GWASs) have identified a strong link between SNPs on chromosome 9 (9p21.3), adjacent to the *INK4/ARF* locus, and atherosclerotic diseases [108]. CircRNA circANRIL, derived from this SNP, disrupts rRNA splicing by binding to the 60S translational assembly factor PES1 and eventually provides promising therapeutic targets for atherosclerosis [109]. A circRNA hsa_circ_0030042 inhibits abnormal autophagy in HUVECs and improves atherosclerosis in vivo by recruiting eukaryotic initiation factor 4A-III (eIF4A3) [110]. Conversely, suppressing circ_0086296 in endothelial cells prevents the formation of atherosclerotic lesions by modulating the repression of miR-576-3p [111]. Cell communication also plays an important role in the progression of atherosclerosis. CircRNA CDR1as can be transferred through exosomes to endothelial cells, activate the FUS (fused in sarcoma)-phos-p65 axis, and eventually promote endothelial dysfunction and aggravated atherosclerosis.

**Figure 3:**
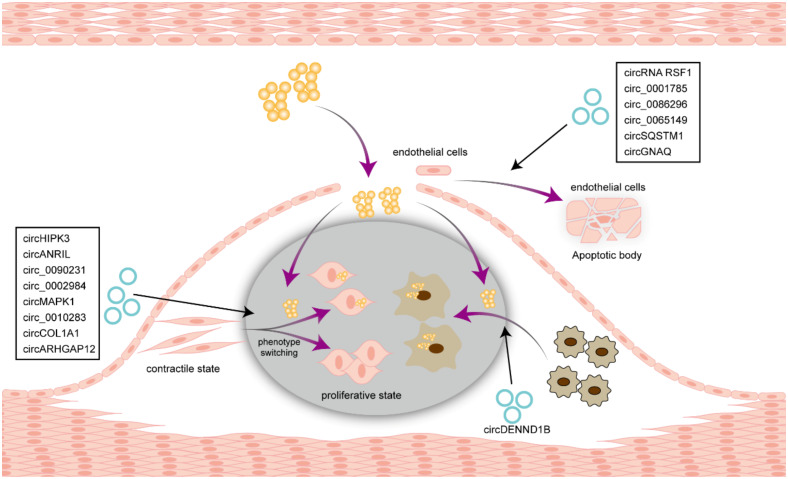
The role of circRNA in atherosclerosis. In atherosclerosis, circRNAs modulate the phenotype-switching process of VSMC, endothelial cells apoptosis and the transforming of macrophages to foam cells.

Oxidized low-density lipoproteins (ox-LDLs) are a key risk factor for atherosclerosis, and influence the physiological function of smooth muscle and endothelial cells [112,113]. Several circRNAs have been reported to be induced by ox-LDL in VSMC or HUVECs, such as circRSF1 [114], circ_0086296 [115], Circ_0090231 [116], circ_0002984 [117], and circ_0010283 [118] (Figure 3 and Table 1), which regulate the proliferation and migration of VSMC or HUVECs and are then expected to serve as biomarkers and therapeutic targets for atherosclerosis.

### 6.2. Arterial Injury

**Figure 4:**
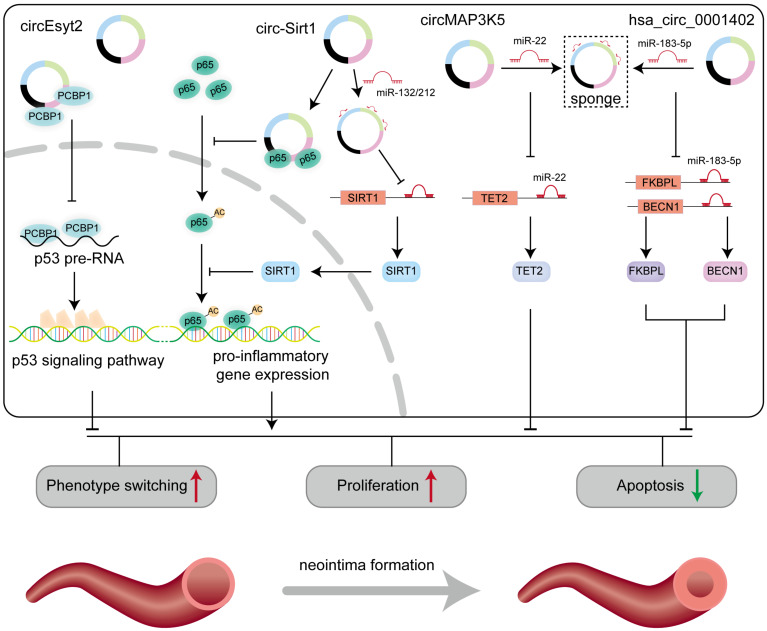
The roles of circRNA in vascular injury. When the artery is injured, smooth muscle cells undergo a phenotypic switch from a contractile state to a proliferative state. In this process, many circRNAs play a critical role, including circEsyt2, circ-Sirt1, circMAP3K5 and has_circ_0001402. ↑ means upregulation, while ↓ means downregulation.

Arterial injury generally occurs after coronary interventional surgery. Stents may damage the inner layer of blood vessels, which impairs endothelial cells and VSMCs, subsequently promoting neointima formation and vascular restenosis [151,152]. Currently, stents are generally coated with drugs that inhibit cell proliferation to mitigate vascular intima proliferation in post-obstruction restenosis [153]. A large population-based study in Germany demonstrated that drug-eluting stents significantly reduce the incidence of postinterventional arterial injury compared with bare metal stents [154]. However, more effective drugs are still necessary to reduce vascular stenosis after arterial injury. Circ-Sirt1 is stably expressed in vascular smooth muscle cells (VSMCs) and is derived from the Sirt1 gene and the TNFα signaling pathway. Mechanism studies showed that circ-Sirt1 can bind to NF-κB p65 and inhibit the transcription of regulatory genes in the nucleus. Meanwhile, circ-Sirt1 acts as a sponge for miR-132/212 to promote the expression of SIRT1 and further inhibit the activity of NF-κB p65. Subject to the activity of the two mechanisms, circ-SIRT1 inhibits the function of VSMCs and the formation of vascular intima [140]. CircRNA circ_LRP6 adsorbs miR-145, promotes vascular smooth-muscle cell phenotype switching, and significantly inhibits arterial intimal proliferation to avoid in-stent restenosis [155]. Induced by platelet-derived growth factor-BB (PDGF-BB), circMAP3K5 sequesters miR-22-3p to facilitate the expression of TET2 (ten-eleven translocation-2), and provide a potential therapeutic strategy for diseases associated with intimal hyperplasia [136]. As shown in Figure 4 and Table 1, these studies on arterial injury have deeply explored the role of circRNAs and are expected to be used as potential targets to develop drugs to prevent vascular occlusion.

### 6.3. Aortic Aneurysm or Dissection

Aortic aneurysm or dissection is an urgent CVD that poses a serious threat to the lives of patients [156]. Surgical intervention is the main strategy for the treatment of an aortic aneurysm or dissection, as there are no specific drugs that can effectively prevent the expansion of the aortic aneurysm or the rupture of the aortic dissection [157,158]. Therefore, elucidating the molecular mechanisms that underlie these diseases is vital for developing potential treatments. Researchers have identified specific circRNAs associated with aortic aneurysm (AAA) by studying macrophages at different polarization stages. One of these circRNAs, circCdyl, was found to be positively correlated with AAA. CircCdyl inhibits the nuclear translocation of interferon regulatory factor 4 (IRF4) and sponges let-7c to promote C/EBP-δ expression, leading to M1 polarization and accelerated AAA formation [141]. Furthermore, a study involving RNA sequencing from human aortic aneurysm tissue and normal aortic tissue identified 411 differentially expressed circRNAs [159]. Of these, circCBFB adsorbs miR-28-5p to regulate the expression of LYPD3 and GRIA4 and promote the progression of aortic aneurysm [144]. Additional circRNAs, including circCCDC66 [147] and circ_0020397 [149], are also involved in the progression of aortic aneurysm or dissection. These studies demonstrate the novel molecular mechanism of circRNAs in aortic aneurysm or dissection, offering a new perspective for the treatment of aortic aneurysm or dissection.

### 6.4. Myocardial Infarction (MI)

The biological activity of cardiomyocytes makes them important contributors to the pathological process of MI, including apoptosis and regeneration of cardiomyocytes, activation of quiescent cardiac fibroblasts and of inflammatory cells [160,161]. Numerous researchers have attempted to clarify the underlying mechanism of MI from the perspective of pathological processes [162]. During the acute phase of MI, cardiomyocyte death occurs rapidly as a result of hypoxia, including cell death by apoptosis, ferroptosis, and pyroptosis. CircRNA circSNRK reduces cardiomyocyte apoptosis by sponging miR-103-3p and then increasing SNRK protein [163]. CircRNA FEACR overexpression represses cardiomyocyte ferroptosis and protects heart function against ischemia/reperfusion (I/R) injury by mediating the NAMPT/Sirt1-/FOXO1/FTH1 signaling axis [164]. Circ-NNT promotes cardiomyocyte pyroptosis and myocardial I/R injury by regulating USP46. Therefore, the activities of circRNA have non-negligible roles in myocardial death and in the early phases of MI [165].

The proliferation and regeneration of cardiomyocytes can maximize the recovery of the damaged myocardium and cardiac function. Thus, many studies have focused on illustrating the underlying mechanism of cardiomyocyte proliferation to develop therapeutic targets. By integrating circRNA high-throughput sequencing with H3K27ac ChIP sequencing, researchers discovered that circNFIX, regulated by a super enhancer, promotes YBX1 degradation via the ubiquitination–proteasome pathway, inhibits the expression of proliferative genes, and subsequently suppresses cardiomyocyte proliferation [166]. Furthermore, the cardiac circRNA expression profile identified circMap4k2 as the most upregulated circRNA, enhancing cardiomyocyte proliferation and cardiac regeneration by targeting the miR-106a-3p and the antizyme inhibitor 1 (Azin1) gene after surgical ventricular reconstruction [167].

Mitochondrial metabolism disorder is a critical alteration resulting from hypoxia-induced MI [168,169]. Overexpression of circSamd4 induced Vcp protein to be translocated into mitochondria, decreased oxidative stress production, maintained mitochondrial dynamics, and improved cardiac function [170]. Additionally, circRNA circ-SNRK targets miR-33 to boost ATP synthesis and improve cardiac function post myocardial infarction [163].

Acute MI causes massive damage to the coronary microcirculation, leading to reduction in vascular and capillary capillaries in the infarct area [171,172]. Therefore, how to construct revascularization is essential to restore cardiac function after MI. CircRNA circFndc3b binds to Fus to regulate *VEGF* expression and enhance the regeneration of blood vessels after myocardial injury [173]. CircNIFX can also sponge miR-214 to promote Gsk3β expression and repress β-catenin activity, eventually repressing angiogenesis [166]. CircERBB2IP can promote angiogenesis after MI through the miR-145a-5p/Smad5 axis [174].

**Figure 5:**
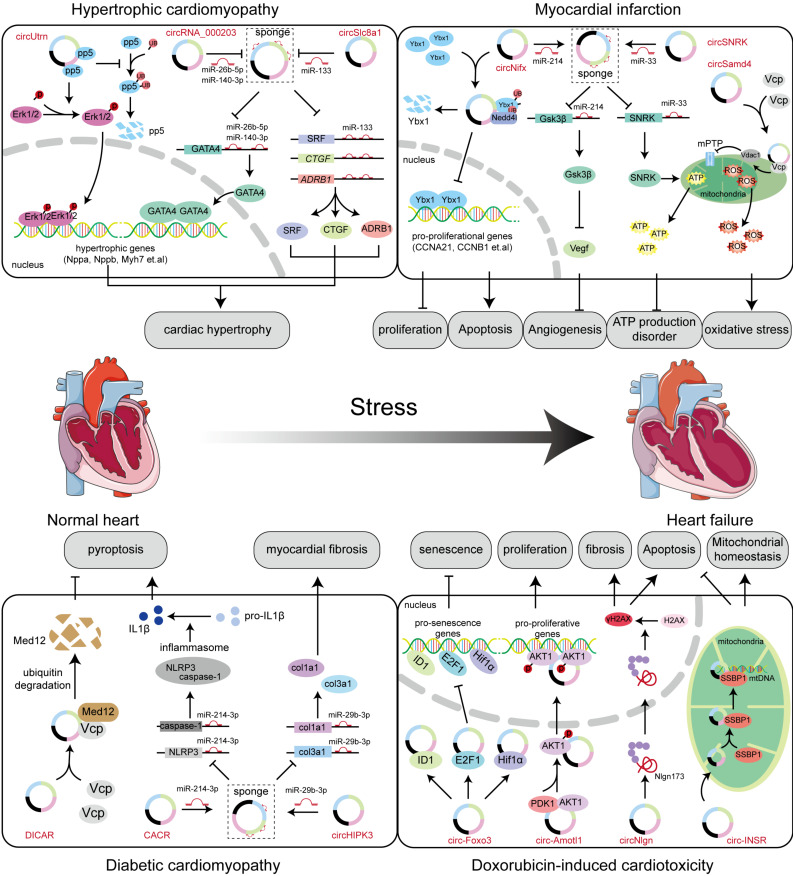
The roles of circRNAs in cardiac diseases. In hypertrophic cardiomyopathy, circUtrn can maintain the stability of pp5 by protecting it from ubiquitin–protease degradation, then activate Erk1/2 and carry out its positively transcriptional function: as a miRNA sponge, and circRNA_000203 and circSlc8a1 targeted miR-26b-5p, miR-140-3p and miR-133 having their pro-hypertrophic effect. In myocardial infarction, circNfix promoted the degradation of Ybx1 and deactivated its pro-proliferative capacity. In addition, circNfix sponged miR-214 to repress angiogenesis. circSNRK repressed its parent genes by sponging miR-33, and then promoted energy production. circSamd4 interacted with Vcp to enhance oxidative stress. In diabetic cardiomyopathy, circRNA DICAR interacted with Vcp and mediated the ubiquitin–protease degradation of Med12, and then inhibited cardiomyocytes pyroptosis. circRNA CACR and circHIPK3 could act as a miRNA sponge to bind miR-214-3p and miR-29b-3p to play their pyroptosis effect and myocardial fibrosis. In doxorubincin-induced cardiotoxicity, circ-Foxo3 bound to ID1, E2F1, Hif1α to repress their nucleus translocation, and then inhibited cardiomyocytes senescence. Circ-Amotl1 promoted AKT1 phosphorylation and its nucleus translocation, eventually enhancing cardiomyocyte proliferation. circNlgn could translate the peptide Nlgn173. The peptide in the nucleus enhanced the transforming of H2AX to γH2AX, and promoted cardiomyocytes apoptosis and fibrosis. circ-INSR in mitochondrial interacted with SSBP1 and maintained the stability of mtDNA, and eventually repressed cardiomyocyte apoptosis and enhanced mitochondrial homeostasis.

As shown in Figure 5 and Table 2, these findings show us the complex regulatory mechanisms in MI and deepen our understanding of the underlying mechanisms of the occurrence and progression of MI.

### 6.5. Hypertrophic Cardiomyopathy

Hypertrophic cardiomyopathy is the most common hereditary heart disease, with a high risk of sudden cardiac death, and which occurs most frequently in adolescents, causing serious social burden and problems [233]. Exploring an effective strategy to prevent the progression of hypertrophic cardiomyopathy is critical to reducing sudden cardiac death. CircSlc8a1, found in cardiomyocytes, can accentuate cardiac hypertrophy and heart failure when overexpressed [185]. Furthermore, a study published in the *New England Journal* explored the regulatory mechanisms of circRNA in hypertrophic cardiomyopathy, revealing that HRCR circRNA reverses the regulatory effect of miR-223 on the downstream gene ARC and ameliorates myocardial hypertrophy and heart failure [181]. Furthermore, plasma circDNAJC6, circTMEM56, and circMBOAT2 can distinguish hypertrophic cardiomyopathy from healthy individuals, offering potential new targets for clinical diagnosis [234]. In cardiac tissue from patients with hypertrophic cardiomyopathy, circZFPM2 was selected using data from global circRNA profiling. Further studies demonstrated that silencing circZFPM2 expression induced cardiomyocyte hypertrophy, compromised mitochondrial respiration, and led to an increase in reactive oxygen species levels [235] (Figure 5 and Table 2).

### 6.6. Dilated Cardiomyopathy (DCM)

DCM is characterized as a disease of the myocardium, with structural and functional abnormalities that induce ventricular dilatation or systolic dysfunction and eventually lead to heart failure and life-threatening arrhythmias [236,237]. Although the role of genetic factors in dilated cardiomyopathy is crucial, other factors, such as infection and immune-related diseases, can also lead to the occurrence of dilated cardiomyopathy [236]. Therefore, many studies have attempted to elucidate the pathogenesis of diabetic cardiomyopathy from the perspective of circRNA. By circRNA sequencing in human heart and the experimental validation in DCM compared with normal healthy sample, 43 circRNAs were identified, such as circSLC8A1, circCAMK2D, and cTTNs, which could be generated by RBM20, an RNA-binding protein and a splicing repressor [238]. To more comprehensively elucidate the role of circRNAs in DCM, another study directly detected heart tissues in patients with DCM by circRNA sequencing and identified 298 differentially expressed circRNAs, in which 213 circRNAs were upregulated and 85 circRNAs were downregulated. They also used bioinformatics to construct a ceRNA-based circRNA-miRNA-mRNA network, which sheds light on the pathogenesis of DCM from the perspective of circRNAs [239].

### 6.7. Diabetic Cardiomyopathy

The high incidence and trend for diabetes in younger people pose a serious threat to health. Diabetic cardiomyopathy, as the most serious complication of diabetes, serves as the main cause of mortality in later stages of the disease [240,241,242]. Therefore, many researchers have attempted to explore the pathogenesis of diabetic cardiomyopathy to find a new therapeutic strategy. Recent interest has surged in circRNAs, notably following discoveries about circRNA DICAR, which improves cardiomyocyte hypertrophy, reduces cardiac fibrosis, and mitigates cardiac dysfunction triggered by diabetic cardiomyopathy. Interestingly, the synthetic junction part of DICAR demonstrates a similar efficacy in improving diabetic cardiomyopathy as the full-length molecule, suggesting a robust basis for its clinical use [231]. CACR, another circRNA, shows elevated expressions in myocardial cells exposed to high glucose and in the plasma of patients with diabetic cardiomyopathy. Mechanistic studies have revealed that CACR interacts with the pivotal regulatory miR-214-3p, influencing the expression of caspase1 in diabetic cardiomyopathy [226]. These results demonstrate that circRNA is promising for presenting a new potential therapeutic target for diabetic cardiomyopathy (Figure 5 and Table 2).

### 6.8. Doxorubicin (DOX)-Induced Cardiotoxicity

DOX is a widely used chemotherapeutic drug for the treatment of tumors, but its clinical application is limited, due to the life-threatening cardiotoxicity it can cause, including cardiac damage and heart failure [243,244]. Reducing DOX-induced cardiotoxicity is crucial to optimizing therapeutic regimens and lowering mortality rates. To solve this problem, researchers have turned their attention to circRNA. Currently, many studies have helped elucidate the mechanisms of circRNA in DOX-induced cardiotoxicity, revealing the potential to become therapeutic targets for clinical disease. The highly species-conserved circRNA insulin receptor (Circ-INSR) interacts with the single-stranded DNA binding protein (SSBP1) to prevent doxorubicin-mediated cardiotoxicity in vitro and in vivo [222]. CircRNA CircITCH has been found to be downregulated in the injured heart in DOX-induced cardiomyopathy models and acts as a miR-330-5p sponge, thus alleviating DOX-induced cardiomyocyte injury and dysfunction [223] (Figure 5 and Table 2).

## 7. Application of circRNAs in CVDs

### 7.1. CircRNA as a Biomarker

The unique properties of circRNA, such as the lack of free ends and resistance to exonuclease digestion, make it more stable and long-lived than linear RNA. This characteristic makes circRNA a suitable biomarker for predicting the onset and severity of diseases.

Exosomes are the most important form of biomarkers and are widely present in plasma, carrying a variety of substances, including miRNAs and circRNAs. Therefore, several studies have attempted to identify circRNAs to act as biomarkers and then predict the occurrence and development of diseases. In patients with coronary heart disease (CHD), isolated serum exosomes exhibited decreased levels in circ_0001785 [125]. Further, IgE-stimulated macrophage modulated endothelial dysfunction by secreting exosomes carrying circRNA CDR1as [111]. Circ_0086296 was not regulated in exosomes of ECs treated with ox-LDL and patients with atherosclerosis [115]. A clinical trial demonstrated that the level of hsa_circRNA_0001599, identified using the RNA-seq-based approach, was positively correlated with the National Institutes of Health Stroke Scale scores and infarct volumes. By performing ROC analysis to hsa_circRNA_0001599 in LAA-stroke, the researchers observed an area under the curve of 0.805, which declared the circRNA biomarker function of hsa_circRNA_0001599 in LAA-stroke diagnosis [245]. These studies provide novel insight into the roles of circRNA as potential diagnostic and therapeutic targets in atherosclerosis.

In patients with arterial aneurysm, a circRNA hsa_circ_0007990 was identified by comparing circRNA microarray data using peripheral blood samples from participants with unruptured intracranial aneurysms (UIAs) with saccular aneurysm wall enhancement (AWE), UIA without AWE, and healthy controls. Hsa_circ_0007990 had the potential to serve as a blood biomarker for UIA with AWE [246]. Another study also revealed that circDUS2 could act as a potential circRNA biomarker for intracranial aneurysm using a human circRNA microarray comparing superficial temporal arteries and intracranial aneurysms [247].

Identifying and evaluating the risk factors associated with developing cardiac remodeling and dysfunction after acute MI is challenging, due to the lack of available biomarkers. To solve this clinical problem, researchers collected peripheral blood samples of patients with acute MI from two independent cohorts and identified circRNA MICRA using an in silico approach. MICRA levels were decreased in patients with MI compared with those in healthy volunteers from the test cohort. Furthermore, univariate and multivariate analyses based on logistic regression revealed that MICRA had a strong potential to serve as a predictor of left ventricular dysfunction in the test cohort [206]. Another study investigating the forensic diagnosis of sudden cardiac death (SCD) caused by acute ischemic heart disease (IHD) identified circSLC8A1 as having high sensitivity and specificity for the diagnosis of MI and exhibited a positive correlation with creatine kinase MB and degree of myocardial infarction. Another circRNA circNFIX was increased in the early stage of MI and subsequently declined, indicating ischemic myocardial damage. Therefore, combining circSLC8A1 and circNFIX expression may be a better strategy to distinguish IHD-related SCDs [216].

Cardiovascular dysfunction caused by diabetes is the leading cause of death. Therefore, developing potential early biomarkers and providing novel therapeutic strategies for chronic diabetic complications are crucial to prevent disease progression. One study found that circHIPK3 increased significantly in the heart with diabetic cardiomyopathy and so has great potential to become a biological marker and therapeutic target for diabetic complications [248]. In general, the applications of circRNAs as biomarkers may be a promising strategy for the diagnosis of human diseases.

### 7.2. Therapeutic Role of CircRNAs

The proponents of circRNA technology anticipate that circRNAs will become the RNA platform of choice for the pharmaceutical industry and may unlock the emergence of next-generation vaccines, treatments for rare diseases, cancer drugs, and other products. Considering its specific properties, such as high stability, low immunogenicity, and protein/peptide-coding capacity, therapeutic platforms based on circRNAs may have more outstanding application prospects in diseases [249]. Given the global health crisis caused by COVID-19 and the serious consequences resulting from its easy transmission and infection over the past years, researchers have been analyzing the characteristics of the virus and developing circRNA vaccines to treat SARS-CoV-2 and its emergent variants, based on its conserved sites. The SARS-CoV-2 circRNA vaccine (VFLIP-X) encodes a spike protein VFLIP-X, which includes six substituted amino acids to broadly neutralize new variants [250]. Another circRNA vaccine against SARS-CoV-2 encodes a trimeric RBD antigen that induces effective neutralizing antibodies to enhance a sustained humoral immune response [251].

In addition to its application in COVID-19, researchers are also exploring the use of circRNA in CVDs. They have engineered a circRNA (circmiRs) to target known cardiac pro-hypertrophic miR-132 and -212. Specific in vivo delivery of optimized circmiRs to cardiomyocytes attenuated left ventricular hypertrophy and cardiac dysfunction [191]. Despite this application in cardiac hypertrophy, the applications of circRNA in CVDs still have a long way to go.

## 8. Limitations and Outlook

CircRNAs were initially considered a “by-product” of the shearing process when they were first discovered, and were not highlighted by researchers. Advancing research, however, revealed that circRNAs exhibited structural characteristics that differed from those of other noncoding RNAs, highlighting their unique regulatory role and superior therapeutic potential in the pathophysiological process of disease. In this review, we provide a comprehensive overview of the biological function of circRNAs and their regulatory role in CVDs. However, research is merely beginning to unravel functions and specific roles in the cardiovascular system of numerous circRNA molecules. Many challenges still remain in understanding the biogenesis, function, and therapeutic applications of circRNA.

With regard to the biogenesis of circRNAs, despite having illustrated some mechanisms to explain the production of circRNAs, an understanding of the responsible mechanisms still remains limited. Some studies have shown that several transcriptional factors can regulate the formation of circRNA, but these do not affect the linear expression of RNA, such as Mexis [166]. The specific roles of these transcription factors are currently not fully understood. In addition, many studies claim that circRNAs play a key role in CVDs, but we cannot accurately evaluate the actual effects of circRNAs, which poses important limitations on the selection of circRNA for evaluation in clinical trials.

There are also several challenges to overcome before applications of circRNA can be achieved. First, the technology for circRNA cyclization and purification needs further improvement. Currently, some cyclization methods have been developed, such as T4 DNA ligase and T4 RNA ligase [252,253,254], but many researchers debate the efficiency and potential for widespread application. In addition, the circRNA purification methods still require further study. Second, delivery vehicles for circRNA are insufficient. Many studies have concluded that nanoparticles may be a good vehicle to carry drugs and deliver them to the injury site [255,256]; however, the low delivery efficiency and safety of circRNA nanoparticles limit their further application [255,257]. Third, circRNAs are considered less immunogenic than linear RNA, whereas exogenous synthetic circRNAs can activate the immune system in vivo [258]. Therefore, how to reduce the immunogenicity of exogenously synthesized circRNAs remains a major challenge. Additionally, though the roles of circRNAs in the occurrence and development of diseases and their clinical relevance to diseases have been clarified, current research is still at the early stage of clinical application. Limited progress has been made on identifying cirRNAs as targets for the diagnosis and treatment of CVDs.

However, the huge therapeutic potential of circRNA cannot be ignored, and in-depth research on the role of circRNA in diseases and elucidating the huge regulatory network of circRNA remains a key focus of future studies on circRNAs.

## Figures and Tables

**Table 1:** CircRNAs associated with vascular diseases.

CircRNA	Expression	Targeting Cells	Functions and Mechanisms
Atherosclerosis			
circ_0030042 [110]	↓	HUVECs	Inhibiting HUVECs autophagy; sponging eIF4A3
circHIPK3 [119]	↑	HUVECs	Activating autophagy; targeting miR-190b/ATG7 axis
circRSF1 [114]	↓	HUVECs	Inducing cell proliferation, inhibited apoptosis and inflammation; modulating miR-135b-5p/HDAC1 axis
circSCRG1 [120]	↑	HUVECs	Promoting angiogenesis; targeting miR-1268b/NR4A1 axis
circZBTB46 [121]	↑	HUVECs; HCASMC	Increasing the atherosclerotic plaque area; interacting with hnRNPA2B1 and regulating the PTEN/AKT/mTOR pathway
circ_0008896 [122]	↑	VSMC	Enhancing proliferation, migration; sponging hsa-miR-633/CDC20B axis
circDLGAP4 [123]	↑	HUVECs	Promoting cell proliferation; targeting miR-134-5p/PTPN4
circRNA-0006896 [124]	↑	HUVECs	Enhancing proliferation, migration; targeting miR-1264/DNMT1
circ_0001785 [125]	↑	HUVECs	Reducing endothelial cell injury; targeting miR-513a-5p/TGFBR3
CDR1as [111]	↑	HUVECs	Promoting the endothelial adhesion function; targeting FUS-phos-p65
circHIPK3 [126]	↑	VSMC	Promoting cell growth; targeting miR-637/CDK6
circ_0086296 [115]	↑	HUVECs	Promoting ECs injury; sponging miR-576-3p/IFIT1-STAT1 axis
circ_0065149 [127]	↓	HUVECs	Promoting the migration and invasion of HUVECs; targeting miR-330-5p
circANRIL [109]	↑	VSMC	Resulting in the induction of apoptosis; binding to PES1
circ_0090231 [116]	↑	VSMC	Promoting proliferation and migration; sequestering miR-942-5p/PPM1B
circ_0002984 [117]	↓	VSMC	Promoting VMSC viability, migration and inflammation; modulating miR-326-3p and VAMP3
circMAPK1 [128]	↑	VSMC	Promoting proliferation and migration of VSMCs; targeting miR-22-3p/MECP2 axis
circSQSTM1 [129]	↑	HUVECs	Reducing inflammation, and promoting autophagy; sponging miR-23b-3p/Sirt1; interacting with eIF4A3
circ_0010283 [118]	↑	VSMC	Promoting cell viability and migration; targeting miR-370-3p/HMGB1
circCOL1A1 [130]	↑	VSMC	Promoting VSMC phenotype switch; targeting miR-30a-5p/SMAD1
circARHGAP12 [131]	↑	MASMC	Promoting VSMC phenotype switch; binding with miR-630
circDENND1B [132]	↑	RAW264.7	Alleviating foam-cell formation; targeting miR-17-5p/Abca1 Axis
circGNAQ [133]	↓	HUVECs	Reducing endothelial cell senescence, enhancing cell proliferation and angiogenesis; targeting miR-146a-5p-PLK2
circHIF1α [134]	↓	HUVECs	Reducing ox-LDL-induced disabilities of endothelial proliferation; targeting miR-199a-5p/SIRT1 axis
Vascular injury			
circEsyt2 [135]	↑	VSMC	Enhancing cell proliferation and migration and inhibiting apoptosis and differentiation; interacting with PCBP1
circMAP3K5 [136]	↓	VSMC	Inhibiting VSMCs proliferation; targeting miR-22-3p/TET2 axis
Hsa_circ_0001402 [137]	↓	VSMC	Inhibiting VSMC proliferation and migration; activating VSMC autophagy; acting as a miR-183-5p sponge
circSOD2 [138]	↑	VSMC	Blocking SMC proliferation; sponging miR-206/NOTCH3
circDcbld1 [139]	↑	VSMC	Promoting VSMC phenotype switch; targeting miR-145-3p/Nrp1
circ-Sirt1 [140]	↓	VSMC	Repressing VSMC inflammatory response; interacting with NF-κB p65; binding to miR-132/212
Arterial aneurysm			
circCdyl [141]	↑	macrophages	Promoting M1 polarization and M1-type inflammation; inhibitingIRF4 entry into the nucleus; acting as a let-7c sponge
circChordc1 [142]	↓	VSMC	Facilitating the VSMC phenotype; binding to vimentin and ANXA2
circRanGAP1 [143]	↑	HUVECs	Targeting miR-183-5p/miR-877-3p/MPO axis
circCBFB [144]	↓	VSMC	Repressing VSMC apoptosis; serving as a sponge of miR-28-5p
hsa_circ_0031608 [145]	↑	VSMC	Promoting the migration and proliferation capacity of VSMCs
hsa_circ_0087352 [146]	↑	VSMC	Inducing VSMC apoptosis; adsorbing hsa-miR-149-5p
circCCDC66 [147]	↓	VSMC	Inducing proliferation facilitation; sponging miR-342-3p
cATM [148]	↑	VSMC	Enhancing oxidative stress
circ_0020397 [149]	↓	VSMC	Promoting VSMC viability; targeting miR-502-5p/GREM1 axis
circ_0022920 [150]	↓	VSMC	Inhibiting HASMC proliferation and migration; sponging microRNA-650

**Table 2:** circRNA associated with cardiac diseases.

circRNAs	Expression	Functions and Mechanisms
Hypertrophic cardiomyopathy		
circrna_000203 [175]	↑	Aggravating cardiac hypertrophy; suppressing mir-26b-5p-mir-140-3p/Gata4
circUtrn [176]	↑	Preventing acute myocardial injury and pathological cardiac remodeling; binding to PP5
circrna_0068481 [177]	↑	Promoting right ventricular hypertrophy; sponging mir-646, mir-570 and mir-885
circHIPK3 [178]	↑	Inhibiting cardiac hypertrophy and dysfunction; sponging mir-185-3p
circ_0001006 [179]	↑	Aggravating cardiac hypertrophy; sponging mir-214-3p/PAK6
circCMISS1 [180]	↑	Activating the ferroptosis and promoting cardiac hypertrophy; interacting with EIF4A3
HRCR [181]	↓	Inhibiting cardiac hypertrophy and heart failure; sponging mir-223/ARC axis
circ-Ddx60 [182]	↑	Aggravating cardiac hypertrophy; binding and activating eef2
circ_0001052 [183]	↑	Promotes cardiac hypertrophy; sponging mir-148a-3p and mir-124-3p
circ-SH3RF3 [184]	↓	Inhibiting myocardial fibrosis; interacting with GATA4
circSlc8a1 [185]	↑	Promoting cardiac hypertrophy; sponging mir-133a
circCACNA1c [186]	↑	Promoting pathological hypertrophy; binding to mir-29b-2-5p
ca-circSlc8a1 [187]	↓	Inhibiting congestive heart failure and maintaining heart function; translocating into mitochondria to drive ATP synthesis
circPAN3 [188]	↓	Attenuating cardiomyocyte hypertrophy; targeting mir-320-3p
circ-SIRT1 [189]	↓	Attenuating autophagy and cardiac hypertrophy; sponging mir-3681-3p/mir-5195-3p and stabilizing SIRT1 protein by recruiting USP22
circ_0018553 [190]	↓	Attenuating Ang II-induced cardiac hypertrophy; sponging mir-4731/SIRT2 signaling pathway
Engineered circular RNA [191]	↓	Attenuating cardiomyocyte hypertrophy; sponging mir-132 and -212
circYAP [192]	↓	Attenuating cardiac fibrosis; binding with TMP4 and ACTG
circITGA9 [193]	↑	Promoting cardiac fibrosis; binding with tropomyosin 3
Myocardial infarction		
circSamd4 [170]	↓	Inducing CM proliferation and preventing CM apoptosis; inducing the mitochondrial translocation of the Vcp protein
circNfix [166]	↑	Inhibiting cardiomyocyte proliferation and angiogenesis and promoting cardiomyocyte apoptosis; interacting with Ybx1; acting as a sponge for mir-214
circUBE3a [194]	↑	Promoting CF proliferation, migration, and phenotypic transformation; exacerbating myocardial fibrosis; sponging mir-138-5p
circFEACR [164]	↓	Inhibiting hypoxia and reoxygenation-induced ferroptosis; mediating NAMPT-Sirt1-FOXO1-FTH1 signaling axis
circHIPK3 [195]	↓	Attenuating cardiac dysfunction and decreasing fibrotic area; binding to Notch1 and mir-133a
circSNRK [163]	↓	Reducing apoptosis and promoting cardiac repair; targeting mir-103-3p/SNRK axis
circWHSC1 [196]	↑	Inducing CM proliferation, alleviating cardiac fibrosis and restoring cardiac function; reinforcing the binding of TRIM59 to STAT3 by enhancing TRIM59 phosphorylation
circPOSTN [197]	↑	Promoting myocardial injury and cardiac remodeling; serving as a mir-96-5p/BNIP3 axis
circ-NNT [165]	↑	Activating pyroptosis; sponging mir-33a-5p/USP46 axis
circFndc3b [173]	↓	Reducing cardiomyocyte apoptosis, enhancing neovascularization; interacting with FUS to regulate VEGF expression
circROBO2 [198]	↑	Promoting myocardial apoptosis; targeting mir-1184/TRADD
circHELZ [199]	↑	Exacerbating myocardial fibroblast proliferation and differentiation; facilitating YAP localization in the nucleus
circ_0001206 [200]	↓	Promoting cell viability and inhibiting cardiomyocyte apoptosis; sponging mir-665 and regulating CRKL expression
circ Foxo3 [201]	↓	Ameliorating cardiac autophagy, apoptosis, inflammation; inhibiting HMGB1 by repressing KAT7
circ-SNRK [202]	↓	Improving the cardiac function by improving the ATP synthesis; sponging mir-33/SNRK axis
circ_0060745 [203]	↑	Promoting macrophage migration and cardiomyocyte apoptosis; activating NF-κB
circMDC1 [204]	↑	Blunting the regenerative capacity of neonatal hearts; binding to PABP
CNEACR [205]	↓	Attenuating myocardial necrosis; binding to HDAC7 and increasing FOXA2 expression
MICRA [206]	↓	Predicting LV dysfunction
circTtc3 [207]	↑	Counteracting hypoxia-induced ATP depletion and apoptotic death; sponging mir-15b-5p/Arl2 axis
circERBB2IP [174]	↓	Promoting CMEC proliferation, migration, and tube formation; targeting mir-145a-5p/Smad5 axis
circSAMD4a [208]	↑	Aggravating H/R-induced cardiomyocyte apoptosis and inflammatory response; sponging mir-138-5p
circrBMS1 [209]	↑	Aggravating hypoxia-induced cardiomyocyte injury; sponging mir-742-3p/FOXO1 axis
circHELZ [210]	↑	Activating NLRP3 inflammasome; sponging mir-133a-3p
circMAP4K2 [167]	↑	Promoting cardiomyocyte proliferation; binding to mir-106a-3p
circPVT1 [211]	↑	Inhibiting cardiomyocyte proliferation and viability, and promoting apoptosis; sponging mir-125b and mir-200a
circPan3 [212]	↑	Increasing cell proliferation and migration; sponging mir-221 through foxo3/ATG7-activated autophagy
MFACR [213]	↑	Promoting mitochondrial fission and cardiomyocyte apoptosis; sponging mir-652-3p and increasing MTP18 expression
ACR [214]	↓	Attenuating myocardial ischemia/reperfusion injury by suppressing autophagy activating Pink1 expression; binding to Dnmt3B and
circCEBPZOS [215]	↓	Attenuating myocardia remodeling by promoting angiogenesis; targeting mir-1178-3p/PDPK1
circSlc8a1/circNfix [216]	↑/↓	Auxiliary diagnostic markers for SCD caused by acute IHD
circ_0002612 [217]	↓	Promoting cardiomyocyte viability; targeting mir-30a-5p/Ppargc1a axis
Myocardial injury		
circ-ZNF609 [218]	↑	Aggravating cardiomyocyte apoptosis, promoting ROS production and increasing iron overload; repressing FTO expression
circ-Ltbp1 [219]	↑	Aggravating DOX-induced proliferation inhibition, inflammation, apoptosis, and oxidative stress; increasing ADCY1 expression by sponging mir-107
circPan3 [220]	↓	Inhibiting DOX-induced myocardial apoptosis
circ-Amotl1 [221]	↓	Protecting against Dox-induced cardiomyopathy; increasing the nuclear fraction of pAKT
circ-INSR [222]	↓	Reducing cardiomyocyte cell death and mitochondrial damage; interacting with SSBP1
circITCH [223]	↓	Alleviating oxidative stress and DNA damage induced by doxorubicin; sponging mir-330-5p
circNlgn [224]	↑	Decreasing cardiac function and inducing cardiac fibrosis; binding and activating H2AX
Quaking Inhibits Doxorubicin-Mediated Cardiotoxicity Through Regulation of Cardiac Circular RNA Expression [43]		
Circ-Foxo3 [225]	↑	Inducing cellular senescence of mefs; interacting with ID-1 and E2F1
Diabetic cardiomyopathy		
CACR [226]	↑	Aggravating DCM pyroptosis; sponging mir-214-3p
CDR1as [227]	↑	Promoting cardiomyocyte apoptosis; activating the Hippo signaling pathway
circHIPK3 [228]	↑	Increasing myocardial fibrosis; sponging mir-29b-3p
circ_0071269 [229]	↑	Increasing myocardial damage; sponging mir-145
circ-Amotl1 [230]	↑	Enhancing cardiac fibrosis; binding with EIF4A3 and stabilizing MARCKS expression
DICAR [231]	↓	Alleviating diabetic cardiomyocyte pyroptosis; binding to VCP
circMAP3K5 [232]	↑	Promoting cardiomyocyte cell apoptosis; sponging mir-22-3p

↑ means upregulation, while ↓ means downregulation.
